# Autism and Epilepsy in Patients With Tuberous Sclerosis Complex

**DOI:** 10.3389/fneur.2020.00639

**Published:** 2020-08-11

**Authors:** Nicola Specchio, Nicola Pietrafusa, Marina Trivisano, Romina Moavero, Luca De Palma, Alessandro Ferretti, Federico Vigevano, Paolo Curatolo

**Affiliations:** ^1^Rare and Complex Epilepsy Unit, Division of Neurology, Department of Neurosciences, Bambino Gesù Children's Hospital, IRCCS, Rome, Italy; ^2^Member of European Reference Network EpiCARE; ^3^Child Neurology and Psychiatry Unit, Systems Medicine Department, Tor Vergata University, Rome, Italy; ^4^Department of Neuroscience, Bambino Gesù Children's Hospital, IRCCS, Rome, Italy

**Keywords:** tuberous sclerosis complex, epilepsy, autism spectrum disorder, prognostic factors, age at onset, genetic, TSC1, TSC2

## Abstract

**Introduction:** Individuals with Tuberous Sclerosis Complex (TSC) are at increased risk of developing both epilepsy and autism spectrum disorder (ASD), but the relationship between these conditions is little understood. We reviewed published reports to elucidate the relationship between ASD, epilepsy, and TSC, and to define the genetic and neurological risk factors.

**Methods:** Articles (January 2004–May 2019) were identified via PubMed, EMBASE, and CENTRAL databases. Article inclusion required report on individuals with TSC-associated ASD and epilepsy with prevalence, odds ratio, or rate report on the comorbidity of ASD in epileptic patients due to TSC.

**Results:** A total of 841 abstracts were identified in the original search. Thirty-six articles were included, which identified study populations, ASD measures used, and study confounders as bias factors. This review included 2,666 TSC patients, with a mean age of 15.9 years (range 1.94–30.3 years). The percentage of TSC patients with epilepsy *and* autism was 33.7%. Patients with TSC *and* autism showed more frequent seizures and earlier epilepsy onset than TSC patients without autism. ASD and intractable epilepsy were both predicted by a higher number of areas with dysplastic features revealed in brain MR scans. ASD, the onset of seizures in children <2 years of age, and >3 tubers have all been associated with an increased risk of refractory epilepsy in TSC patients. However, the direction of the relationship is not clear because a history of epilepsy, or infantile spasms in patients with TSC is also associated with an increased likelihood of ASD. Overall, 73.2% of patients carried *TSC2* genetic variant and, among patients with TSC and autism, the percentage of *TSC2* individuals was 85.6%.

**Conclusions:** The complex interrelationship between TSC, autism, and epilepsy, coupled with limited knowledge on the neurobiological basis for the interrelationship, limits overall understanding and opportunities for management. The results of this review highlight the need for early identification and management to optimize favorable outcomes in the most vulnerable individuals with TSC. Regardless of whether studies are considered individually or collectively, interpretation is made difficult due to the differences between the studies, most notably between methods and diagnostic criteria used to assess intellectual ability.

## Introduction

Tuberous Sclerosis Complex (TSC) is a rare genetic multisystem disorder characterized by hamartoma formation in several organs and systems (1, 2), with an estimated birth incidence of 1 in 5,800 (3). TSC is caused by mutation in either *TSC1* (chromosome 9q34) or *TSC2* (16p13.3) gene, encoding for hamartin and tuberin, respectively (4). These two proteins, along with TBC1D7, form a heterotrimeric complex regulating the activity of mTOR complex 1 (mTORC1), which is a key regulator of cell metabolism and proliferation. mTORC1 dysregulation is the main reason for aberrant growth and differentiation underlying the formation of TSC-related lesions, either in the brain or other organs (1).

Neurologic and developmental issues such as epilepsy, autism spectrum disorder (ASD), and developmental delay (DD), are major sources of morbidity in people of all ages with TSC and typically present in infancy or early childhood (1). Epilepsy is estimated to occur in about 80% of TSC patients, typically within the first 3 years of life, and considered to be a result of the genetic mutation leading to an imbalance between excitation and inhibition of gamma-amino-butyric acid (GABA) receptors. Dysregulation of the neurotransmission of GABA has also been proposed as a neurobiological link between epilepsy and ASD in TSC patients (5).

ASD is an early onset, lifelong, neurobiological disorder characterized by impairments in communication and social interaction along with the presence of restricted and repetitive patterns of behavior, interests or activities, and is prevalent in 1.85% of children aged 8 years (6–8). In the last 5 years, some longitudinal studies have explored the early emerging symptoms and prompt intervention in infants with high familial risk of ASD (9, 10). In contrast, very few studies have addressed this topic in ASD associated with specific syndromes or genetic conditions (11–13).

TSC is one of the major syndromes associated with ASD. The prevalence of ASD in TSC ranges from 26 to 45%, depending on the sample, ASD criteria, and the testing methodologies employed (14, 15). Some autistic features are present in about half of patients with TSC. A number of factors have been identified as being associated with ASD in TSC, including brain lesion load, prominent lesion type, the size and location of the tubers, cyst-like tubers, *TSC2* mutation, early onset and refractory seizures, and the presence and severity of cognitive impairment (1, 16). Prompt cessation of early seizures can, in at least some cases, improve neuropsychiatric outcome (17, 18).

To our knowledge, no review has yet examined the relationship between ASD and epilepsy in patients with TSC. We performed a review of the literature to assess the prevalence and risk factors for ASD in patients with TSC and epilepsy, and to investigate the relationship and comorbidity between these conditions. The main aims of this review were: to identify the frequency of both ASD and epilepsy within the TSC population, and to elucidate the relationship between ASD and epilepsy in individuals with TSC.

## Methods

The results of the present review were reported according to the preferred reporting items for reviews and meta-analyses (PRISMA) and adheres to a structured review protocol (19).

### Search Strategy and Article Selection

Two authors (NP and NS) performed a search of PubMed, EMBASE, and CENTRAL databases using the following search strategy: “autism” OR “autistic” OR “asperger” OR “autism spectrum disorder” OR “pervasive” OR “pervasive developmental disorder” OR “PDD” OR “ASD” AND “epilepsy” OR “seizure” OR “epileptic” OR “convulsion” AND “tuberous sclerosis complex” OR “tuberous sclerosis” OR “TSC.”

Studies were initially included if they:

1. Involved individuals with ASD and epilepsy symptomatic of TSC.
2. Reported prevalence, odds ratio, or numerical report of the comorbidity of ASD in patients with epilepsy due to TSC.
3. Were written in English.
4. Were based on human research.
5. Were published within 15 years of the search date (January 2004–May 2019), which was considered a sufficient period to capture publications with the most reliable and appropriate diagnostic and management procedures.
  Two authors independently screened all titles and abstracts of studies identified by the initial search. The full text of an article was obtained when either reviewer thought that it might fulfill the inclusion criteria. Upon uncertainty for inclusion of a publication, an additional author was consulted (LDP).

Full articles were reviewed for relevance and articles were excluded if they did not include data relating to the prevalence of epilepsy/seizures in the TSC population. Articles also had to contain a reported or calculable prevalence for ASD in the text (if not provided in the abstract).

Based on the Quality in Prognosis Strategy (QUIPS) tool, the most commonly found risk factors for bias in the studies reviewed included study participation, ASD measure, and study confounders. Many [14] of the reviewed articles included participants drawn from one clinic or hospital (18, 20–32); others [5] had a specific age range (12, 13, 33–35) or a particular subset of the TSC population (18, 35–47, 49, 50). Only 18 of the included articles reported the diagnostic criteria for ASD (Table 1). Large variations were noted in the measures and criteria used to define ASD and many of the articles relied on reports of ASD by parents and caregivers. Comparisons between various studies were subject to a number of potential confounders, including a failure to report seizure onset, type, and frequency for epilepsy, antiseizure medication (ASM), genetic susceptibility, or other relevant baseline measures. Only articles that unequivocally reported the above-mentioned information were included in Tables 2–4. From Table 2, eight articles were excluded because of no mention of onset, type, or frequency of epileptic seizures; 16 articles were excluded from Table 3 because of no mention of number of patients with epilepsy, TSC and autism; 14 articles were excluded from Table 4 because of no mention of genetic mutation in *TSC1* and *TSC2*. In this review we have used the terminology “infantile spasms” for infants with ES (with or without hypsarrhythmia), who may or may not have had cognitive regression. This operational definition was chosen because it was not always possible to determine whether the infants had hypsarrhythmia or cognitive regression. In the tables and figures, however, the term “epileptic spasms” has been used because this refers to that specific type of seizure.

**Table 1:** Demographic information and prevalence rates of autism and epilepsy/seizures in Tuberous Sclerosis Complex patients reported within each of the articles included in this review.

**Article**	**Study type**	**TSC patients, *n***	**Male, *n* (%)**	**Mean age (unless median reported)**	**TSC patients with epilepsy/seizures, *n* (%)**	**TSC patients with autism, *n* (%)**	**Autism assessment**	**TSC patients with epilepsy and autism, *n* (%)**
Baumer et al. (36	Retrospective cohort	17	10 (59%)	7.2 y	10 (59%)	5 (29%)	n/r	n/r
Baumer et al. (37	Retrospective clinical records (MRI)	51	31 (61%)	9.25 y	36 (71%)	19 (37%)	DSM IV/V and ADOS	18 (35%)
Benova et al. (38	Prospective imaging	22	13 (59%)	6.3 y	20 (91%)	9 (41%)	ADI-R	9 (41%)
Capal et al. (51	TACERN Prospective longitudinal study	130	68 (52%)	23.3 mo	95 (73%)	Symptoms only studied	AOSI and ADOS-2	n/r
Caylor et al. (39	Exome sequencing in 3 families	3	2 (67%)	16.3 y	3 (100%)	1 (33%)	n/r	1 (33%)
Chopra et al. (40	Cohort	45	22 (49%)	14.8 y	35 (78%)	15 (33%)	n/r	n/r
Chou et al. (20	Cohort MRI	25	14 (56%)	11 y	23 (92%)	5 (20%)	n/r	n/r
Cusmai et al. (41	Retrospective cohort	44	19 (43%)	13.8 y	44 (100%)	13 (30%)	n/r	13 (30%)
de Vries et al. (33	Postal survey	265	106 (40%)	Reported age in groups (<5 and >18 were excluded)	238 (90%)	119 (45%)	n/r	n/r
Doherty et al. (42	Retrospective study	44	21 (48%)	n/r	44 (100%)	9 (20%)	PDD	9 (20%)
Eluvathingal et al. (43	MRI and PET scans of consecutive patients	78	44 (56%)	8 y	78 (100%)	Symptoms only studied	Gilliam Asperger's Disorder Scale (GADS)^*^ and VABS	n/r
Gül Mert et al. (21	Case study of clinical records	83	43 (53%)	33.5 mo	83 (100%)	28 (34%)	n/r	28 (34%)
Huang et al. (22	Medical records	32	16 (50%)	n/r	26 (81%)	6 (19%)	n/r	n/r
Iscan et al. (23	Brain imaging	17	10 (59%)	9.5 y	15 (88%)	1 (6%)	n/r	0
Jeste et al. (13	Longitudinal study	36	22 (62%)	32.1 mo	34 (94%)	18 (50%)	ADOS	18 (50%)
Kilincaslan et al. (44	Case study of patients with refractory epilepsy	6	4 (67%)	16.25 y^a^	6 (100%)	3 (50%)	CARS and AuBC	3 (50%)
Kingswood et al. (46	Retrospective longitudinal cohort	334	157 (47%)	30.3 y	257 (77%)	41 (13%)	n/r	n/r
Kopp et al. (28	Clinical records	99	45 (45%)	7.7 y	87 (88%)	31 (31%)	n/r	n/r
Kosac and Jovic (25	Retrospective cohort (clinical records)	44	18 (41%)	19.4y	39 (89%)	6 (14%)	n/r	5 (11%)
Metwellay et al. (32	Cross sectional observational study	24	18 (75%)	6.2 y	21 (88%)	11 (46%)	ADI-R and ADOS	n/r
Mizuguchi et al. (45	Randomized trial	29	17 (59%)	8.76 y^a^	29 (100%)	20 (69%)	PARS	20 (69%)
Moavero et al. (34	Epistop prospective study	82	45 (55%)	n/r evaluated at 6, 12, and 18 mo	51 (62%)	25 (30%)	ADOS and BSID	19 (23%)
Muzykewicz et al. (52	Retrospective chart review	241	118 (49%)	20 y	208 (86%)	86 (36%)	Neuropsychological exam or clinical opinion	n/r
Numis et al. (22	Retrospective cohort (clinical records)	103	47 (46%)	13.05 y	91 (88%)	41 (40%)	DSM-IV, Child symptom inventory-4, BASC-2 and Gilliam Asperger's Disorder Scale (GADS)^*^	40 (39%)
Overwater et al. (47	RCT	32	16 (50%)	12 y^a^	25 (78%)	17 (53%)	ADOS and CANTAB	n/r
Pascual-Castroviejo (26	Retrospective review of MRI data	45	23 (51%)	n/r	45 (100%)	16 (36%)	n/r	16 (36%)
Saltik et al. (27	Retrospective study of clinical records	21	11 (52%)	7.5 y	21 (100%)	2 (10%)	DSM-IV	2 (10%)
Samir et al. (35	Prospective EEG and MRI	30	16 (53%)	4.66 y	30 (100%)	12 (40%)	ADIR and ADOS	12 (40%)
Spurling Jeste et al. (12	Prospective study as part of a multisite longitudinal study	40	n/r	data reported at 6 mo intervals	36 (90%)	22 (55%)	AOSI and ADOS	22 (55%)
Staley et al. (49	Retrospective review of clinical records	257	n/r	19 y	210 (82%)	23 (9%)	Gilliam Asperger's Disorder Scale (GADS)^*^	n/r
Toldo et al. (28	Retrospective and prospective cohort study	32	16 (50%)	9.75 y	24 (75%)	22 (69%)	n/r	n/r
Vignoli et al. (29	Cohort Study	42	18 (43%)	19.3 y^a^	42 (100%)	17 (40%)	SCQ	17 (40%)
Wataya-Kaneada et al. (30	Comparison study of current vs. historical data from patients with TSC	166	70 (42%)	26.6 y	138 (83%)	35 (21%)	Pediatric and pychiatric departments (no diagnostic criteria) in Japan	n/r
Wilbur et al. (31	Retrospective review of clinical records	81	41 (51%)	10 y^a^	74 (91%)	20 (25%)	n/r	20 (25%)
Wong and Khong (53	MRI records	22	10 (45%)	15.25 y	21 (95%)	7 (32%)	DSM-IV/ADIR	7 (32%)
Yang et al. (50	Systematic analysis of genotypic and clinical data of Chinese patients	117	60 (51%)	5.17 y	113 (97%)	27 (23%)	n/r	n/r

*ADI-R, The Autism Diagnostic Interview-Revised; ADOS, The Autism Diagnostic Observation Schedule; AOSI, Autism Observation Scale for Infants; AuBC, Autism Behavior Checklist; BASC, Behavioral Assessment System for Children; BSID, Bayley Scales of Infant Development; CARS, Childhood Autism Rating Scale; DSM, Diagnostic Statistics Manual; MRI, magnetic resonance imaging; n/r, not reported; PARS, Pervasive Developmental Disorders Autism Society Japan Rating Scale; PET, Positron emission tomography; SCQ, Social Communication Questionnaire; TSC, Tuberous Sclerosis Complex; VABS, Vineland Adaptive Behavior Scales*.

a *Median age reported*.

* *(54)*.

**Table 2:** Summary of history of epilepsy in patients with Tuberous Sclerosis Complex.

**Article**	**Epilepsy/seizures present in TSC**	**Age at onset, mean**	**Epileptic spasms, *n***	**Epilepsy/seizure type**	**Refractory epilepsy (%)**	**Seizure frequency**
Benova et al. (38)	20	8.1 mo	5	n/r	n/r	Daily (*n* = 14); weekly (*n* = 2); monthly (*n* = 4)
Capal et al. (51)	95	5.6 mo	39	Focal szs (*n* = 21); mixed (*n* = 42) Generalized szs (*n* = 4); unclassified (*n* = 6)	n/r	n/r
Caylor et al. (39)	3	n/r	1	Frontal lobe epilepsy (*n* = 1); Focal szs (*n* = 1);	1 (33%)	n/r
Chou et al. (20)	23	<1 y (*n* = 13); <2 y (*n* = 19)	10	n/r	11 (48%)	n/r
Cusmai et al. (41)	44	<1 y	29	Focal motor szs (*n* = 19); generalized szs (*n* = 1)	14 (32%)	n/r
Doherty et al. (42)	44	n/r	23	n/r	n/r	n/r
Gul Mert et al. (21)	83	25.46 mo	21	Focal (*n* = 23); multifocal (*n* = 12); generalized (*n* = 26)	15 (18%)	n/r
Huang et al. (22)	26	≤ 6 mo (*n* = 11); 7–12 mo (*n* = 8); ≥12 mo (*n* = 4)	7	Complex partial (*n* = 4); simple partial (*n* = 4); generalized (*n* = 7); clonic (*n* = 1); tonic (*n* = 1; myoclonic (*n* = 1)	n/r	n/r
Iscan et al. (23)	15	24.7 mo	4	Generalized (*n* = 3); mixed (*n* = 4); Complex partial (*n* = 2) myoclonic (*n* = 1); febrile (*n* = 1)	n/r	n/r
Jeste et al. (13)	34	5.75 mo	n/r	n/r	6 (18%)	Monthly (26%); weekly (7%); daily (27%)
Kilincaslan et al. (44)	6	<6 mo (*n* = 3); <2 y (*n* = 2); 7 y (*n* = 1)	4	Complex partial (*n* = 2); simple partial (*n* = 2); atonic/atypical absence (*n* = 1)	6 (100%)	>1 a day (*n* = 4); >1 a week (*n* = 2)
Kopp et al. (24)	87	0.9 y	51	Complex partial history (*n* = 78); mixed seizures history (*n* =18)	n/r	Mean per month 39.9 (*n* = 66)
Kosac and Jovic (25)	39	2.8 y	10	Focal szs (84.6%); Secondary generalized szs (39.3%)	n/r	n/r
Metwellay et al. (32)	21	<6 mo (*n* = 12); >6 mo (*n* = 9)	13	Generalized (*n* = 3); Focal (*n* = 4); Partial with secondary generalization (*n* = 1)	16 (76%)	n/r
Mizuguchi et al. (45)	29	n/r	n/r	n/r	n/r	n/r
Moavero et al. (34)	51	<1 y (*n* = 38); <2 y (*n* = 13)	10	n/r	32 (63%)	n/r
Muzykewicz et al. (52)	208	n/r	92	n/r	141 (68%)	n/r
Numis et al. (18)	91	1.9 y	44	n/r	60 (66%)	1.75 per week
Overwater et al. (47)	25	n/r	7	n/r	14 (56%)	n/r
Pascual-Castroviejo et al. (26)	45	n/r	23	n/r	n/r	n/r
Saltik et al. (27)	21	<1 y (76.1%)	5	Focal szs (*n* = 20); diffuse tonic-clonic (*n* = 3); atonic (*n* = 3); absence (*n* = 1)	13 (62%)	n/r
Samir et al. (35)	30	<6 mo (*n* = 16); ≥6 mo (*n* = 14)	17	Focal szs (*n* = 5); secondary generalization (*n* = 8)	19 (63%)	n/r
Spurling Jeste et al. (12)	36	5.8 mo	26	n/r	n/r	n/r
Vignoli et al. (29)	42	7.9 mo	11	n/r	11 (26%)	Monthy (*n* = 7); Weekly (*n* = 10)
Wataya-Kaneada et al. (30)	143	n/r	n/r	n/r	20 (14%)	n/r
Wilbur et al. (31)	74	12 mo median	26	Focal (66%); epileptic spasms (26%); generalized (5%)	n/r	n/r
Wong et al. (55)	21	33 mo	8	n/r	3 (14%)	n/r
Yang et al. (50)	113	n/r	55	n/r	n/r	n/r

*n/r, not reported; Szs, seizures; TCS, Tuberous Sclerosis Complex*.

**Table 3:** Summary of family history of Tuberous Sclerosis Complex (TSC) and genetic mutations in patients with TSC.

**Article**	**TSC patients, *n***	**TSC patients with epilepsy and autism, *n* (%)**	**Seizure/epilepsy in patients with ASD**
Baumer et al. (37)	51	18 (35%)	n/r
Benova et al. (38)	22	9 (41%)	2/9 ES
Caylor et al. (39)	3	1 (33%)	1/1 focal to bilateral seizure
Cusmai et al. (41)	44	13 (30%)	8/13 ES, 5/13 focal motor
Doherty et al. (42)	44	9 (20%)	n/r
Gül Mert et al. (21)	83	28 (34%)	n/r
Iscan et al. (23)	17	0	n/r
Jeste et al. (13)	36	18 (50%)	13/18 ES
Kilincaslan et al. (44)	6	3 (50%)	2/3 ES, 3/3 focal seizure, 2/3 tonic seizure
Kosac and Jovic (25)	44	5 (11%)	n/r
Mizuguchi et al. (45)	29	20 (69%)	n/r
Moavero et al. (34)	82	19 (23%)	2/15 ES
Numis et al. (18)	103	40 (39%)	24/40 ES
Pascual-Castroviejo et al. (26)	45	16 (36%)	n/r
Saltik et al. (27)	21	2 (10%)	n/r
Samir et al. (35)	30	12 (40%)	11/12 ES
Spurling Jeste et al. (12)	40	22 (55%)	14/22 ES
Vignoli et al. (29)	42	17 (40%)	n/r
Wilbur et al. (31)	81	20 (25%)	n/r
Wong and Khong (53)	22	7 (32%)	n/r

*n/r, not reported; TSC, Tuberous Sclerosis Complex; ASD, Autism spectrum disorder; ES, epileptic spasms*.

**Table 4:** Summary of family history of Tuberous Sclerosis Complex (TSC) and genetic mutations in patients with TSC.

**Article**	**TSC pts, *n***	***TSC1* (all patients), *n* (%)**	***TSC1* (patients with autism), *n* (%)**	***TSC2* (all patients), *n* (%)**	***TSC2* (patients with autism), *n* (%)**	**No mutation identified (all patients), *n* (%)**	**No mutation identified (patients with autism), *n* (%)**	**Family history of TSC, *n* (%)**
Benova et al. (38)	22	7 (32%)	2 (22%) (*n* = 9)	12 (55%)	5 (56%) (*n* = 9)	–	–	n/r
Caylor et al. (39)	3	2 (67%)	1 (100%) (*n* = 1)	1 (33%)	0 (*n* = 1)	–	–	n/r
Chopra et al. (40)	45	9 (20%)	1 (7%) (*n* = 15)	24 (53%)	12 (80%) (*n* = 15)	11 (24%)	n/r	5 (11%)
Chou et al. (20)	25	n/r	n/r	n/r	n/r	n/r	n/r	2 (8%)
Cusmai et al. (41)	44	2 (9%) (*n* = 23)	n/r	20 (87%) (*n* = 23)	n/r	1 (4%) (*n* = 23)	n/r	n/r
Doherty et al. (42)	44	10 (23%)	n/r	26 (59%)	n/r	n/r	n/r	n/r
Huang et al. (22)	32	6 (19%)	1 (17%) (*n* = 6)	26 (81%)	5 (83%) (*n* = 6)	–	–	n/r
Iscan et al. (23)	17	n/r	n/r	n/r	n/r	n/r	n/r	4
Jeste et al. (13)	34	5 (16%) (*n* = 31)	4 (27%) (*n* = 15)	26 (84%) (*n* = 31)	11 (73%) (*n* = 15)	–	–	n/r
Kopp et al. (24)	99	15 (16%)	n/r	58 (62%)	n/r	21(22%)	n/r	20 (20%)
Kosac and Jovic (25)	44	3 (30%) (*n* = 10)	n/r	5 (50%) (*n* = 10)	n/r	n/r	n/r	11 (25%)
Moavero et al. (34)	82	20 (24%)	n/r	59 (72%)	n/r	3 (4%)	n/r	n/r
Muzykewicz et al. (52)	241	50^a^ (27%) (*n* = 191)	n/r	106^a^ (55%) (*n* = 191)	n/r	34 (18%) (*n* = 191)	n/r	n/r
Numis et al. (18)	103	24 (23%)	3 (7%) (*n* = 41)	58 (56%)	27 (66%)	10 (10%)	6 (22%) (*n* = 41)	n/r
Overwater et al. (47)	32	7 (22%)	n/r	21 (66%)	n/r	4 (13%)	n/r	n/r
Saltik et al. (27)	21	n/r	n/r	n/r	n/r	n/r	n/r	7 (33%)
Samir et al. (35)	30	n/r	n/r	n/r	n/r	n/r	n/r	4 (13%)
Staley et al. (49)	257	51 (27%) (*n* = 192)	n/r	109 (57%) (*n* = 192)	n/r	n/r	n/r	n/r
Vignoli et al. (29)	42	10 (24%)	n/r	30 (71%)	n/r	2 (5%)	n/r	n/r
Wataya-Kaneada et al. (30)	166	21 (28%) (*n* = 75)	n/r	24 (32%) (*n* = 75)	n/r	30 (39%) (*n* = 75)	n/r	17 (23%) (*n* = 75)
Wilbur et al. (31)	81	2 (33%) (*n* = 6)	n/r	4 (67%) (*n* = 6)	n/r	–	n/r	6 (7%)
Yang et al. (50)	117	16 (14%)	2 (9) (*n* = 27%)	101 (86)	25 (93%) (*n* = 27)	–	–	14 (12%)

*n/r, not reported; TSC, Tuberous Sclerosis Complex; pts, patients*.

a *One patient had both TSC1 and TSC2 mutations*.

## Results

**Figure 1:**
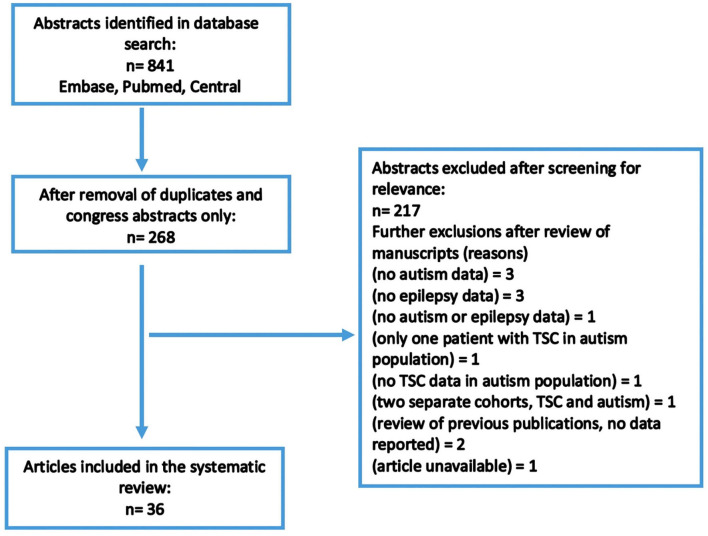
Search strategy.

A total of 841 abstracts were identified in the original search. Of these, 673 were duplicates or congress abstracts only. The remaining abstracts and articles were reviewed for inclusion/exclusion criteria, and a total of 36 articles were considered suitable for inclusion (Figure 1). Included articles are presented in Table 1. In total, 2,666 patients with TSC were included in this review, with a mean age of 15.9 years (range 1.94–30.3 years). TSC populations included within the selected articles were predominantly male; males represented 52.5% of overall participants, ranging from 41 to 75% of patients in articles (Table 1).

### Prevalence of Autism and Epilepsy in Patients With TSC

Of the patients with TSC included with available data in this review, the overall percentage of patients with autism was 29.8% (732 of 2,458 patients with available data), ranging from 6% (23) to 69% (28, 45), and those with epilepsy/seizures was 88.2% (2,352 of 2,666 patients), ranging from 59 to 100% (21, 26, 27, 29, 35, 39, 41–45) (Table 1). Patients with epilepsy *and* autism are also reported where available (Table 1), with the overall percentage being 33.7% (279 of 828 patients with available data) and ranging from 10% (27) to 69% (45).

### Epilepsy

The mean age for onset of epilepsy was below 33 months; however, data were available for 859 patients only. Infantile spasms were reported in 42.8% of TSC populations studied (Table 2), ranging from 20% (34) to 67% (44). Other epilepsy types were less frequently reported within the articles reviewed, but Huang et al. (22) suggested that focal seizures were also frequent in infants with TSC under 1 year of age. Reports of refractory epilepsy in patients with TSC ranged from 14% to 100% (44), although the latter specifically focused on 6 TSC patients with refractory epilepsy.

The relationship between epilepsy, ASD, and TSC is complex. Autism, the onset of seizures in children <2 years of age and with >3 tubers (21, 31) have all been associated with an increased risk of refractory epilepsy in TSC patients. However, the direction of the relationship is unclear because a history of epilepsy (33) or infantile spasms (31, 35) in patients with TSC is also associated with an increased likelihood of ASD. Patients with TSC *and* autism showed more frequent seizures than TSC patients without autism (18) and an earlier age of onset of epilepsy has been associated with ASD (18, 28, 31), delayed language, intellectual disability (ID), and poor cognitive flexibility (28). In Table 3 are reported the epilepsy features in patients with TSC and autism.

### Phenotype/Behavior

Clinically significant behavioral problems and social withdrawal are common in young children with TSC (28). Conditions including mood disorder, anxiety, ADHD, and aggressive behavior were reported in 66% of a pediatric population with TSC (*n* = 241) (52). Aggressive behavior was associated with both increased severity of epilepsy and features of autism/pervasive developmental disorder (PDD) (52).

Early identifiers of autism or autistic-like features in patients with TSC include early DD or a slowing in nonverbal cognition (13, 38). Studies of very young infants with TSC suggest early delay in visual reception (12) and under-developed fine-motor skills to be markers of the development of autism traits (12, 34). Deficits across all domains of the Bayley Scales of Infant Development (BSDI) at 1 year of age were predictive of higher autism traits on the Autism Diagnostic Observation Schedule (ADOS) at 2 years within a prospective study of infants with TSC (*n* = 82) in 10 sites across Europe and Australia (34). ID is often more common in TSC patients with ASD than those with TSC alone (31). Behavioral problems have been reported to be exacerbated by seizure frequency and a mixed seizure profile (24). Results of a study exploring the relationship between cognitive delay and clinical features of TSC in Egypt reported that the age of seizure onset (*p* = 0.044) and number of brain tubers (*p* = 0.06) increased the odds for cognitive delay in 24 children with TSC (32). Similarly, ID has been associated with early onset of seizures, infantile spasms (35), and intractable epilepsy (38). Early seizure onset was the most significant predictor of DD at 2 years of age in a longitudinal prospective analysis of developmental outcomes in infants (0–3 years) with TSC (51). Since the data in most of the reported studies did not specify infantile spasms, many of the early onset seizures could have been infantile spasms. The neurologic symptoms of TSC, refractory epilepsy, ASD, and ID have all shown an interrelationship (30), making specific relationships between ASD, ID, and epilepsy difficult to discern.

Self-injurious behavior in patients with TSC was associated with a history of infantile spasms and seizures, ID, ASD, and *TSC2* mutations (49). Aggressive behavior was also associated with ID and *TSC2* mutations (52), suggesting a potential genetic link.

Data on severity of autism and developmental delay were sparse and therefore not reported.

### Genotype

Refractory epilepsy (38), ID (24), and autism in TSC patients have all been associated with the *TSC2* genotype (29, 40, 50). The *TSC2* genotype was more common than *TSC1* genotype among TSC patients overall (Table 4), with the exception of one study that focused on individuals from three families, in which three individuals had a diagnosis of TSC: two with the *TSC1* genotype and one with the *TSC2* genotype (39). Overall, 73.2% of TSC individuals had the *TSC2* genotype—ranging from 32% (30) to 89% (41)—and 26.8% had the *TSC1* genotype—ranging from 9% (41) to 67% (39) (Table 4). Among patients with TSC and autism, 85.6% had the *TSC2* genotype. Autistic behavior correlated with nonsense mutations in the *TSC2* gene group in a retrospective review of medical records from patients with TSC in Taiwan (*n* = 32) (22).

### Neuroimaging

A magnetic resonance imaging (MRI) study including 25 children (aged >2 years) reported that lesion load within the left temporal lobe was positively correlated with the neurological severity score (*r* = 0.609; *p* = 0.001). This finding was supported in an electroencephalogram (EEG) study that found greater interictal epileptiform features in the left temporal lobe only (18).

Two studies exploring potential impact of TSC proteins on white-matter tract pathways have identified abnormal diffusion characteristics, which are believed to arise from abnormal neuronal and axonal organization and hypomyelination (36, 37). Furthermore, these effects were each associated with TSC, epilepsy, and autism (36, 37). In a diffusion MRI study exploring the directionality of water movement [fractional anisotropy (FA)], TSC alone was related to lower callosal FA values than controls—and this difference was greater in the TSC patients with autism than without—when comparing study groups of TSC patients with either epilepsy (with and without comorbid autism; *n* = 19 and *n* = 32, respectively) or autism alone (*n* = 46) with a healthy control group (*n* = 89) (37). A positron emission tomography (PET) study comparing TSC patients with and without a cerebellar lesion (*n* = 20 vs. *n* = 57, respectively) reported that the group with cerebellar lesions had higher overall autistic symptomology (i.e., social isolation and communicative/developmental disturbance) and that these deficits were associated with right-sided cerebellar lesions (43).

The size, number, and anatomical location of tubers have all independently been linked to autism and/or epilepsy in TSC (22, 26, 35, 38, 42), although this relationship has not always been established (55). The number of tubers is strongly associated with infantile spasms (42) and ASD (35). Tubers of larger size were associated with increased likelihood of seizures and autism (26), and higher prevalence of cyst-like tubers was associated with ASD (22). ASD and intractable epilepsy were both predicted by a higher number of areas with dysplastic features (38). ASD or PDD have been linked with tubers in the frontal areas of the brain (35), increased tuber count in the occipital lobe (42), cystic-like tubers, and tubers in insular and temporal areas (22). Infantile spasms are more likely to occur in children with cortical tubers in the parietal lobes (55).

### Pharmacological Treatment

**Figure 2:**
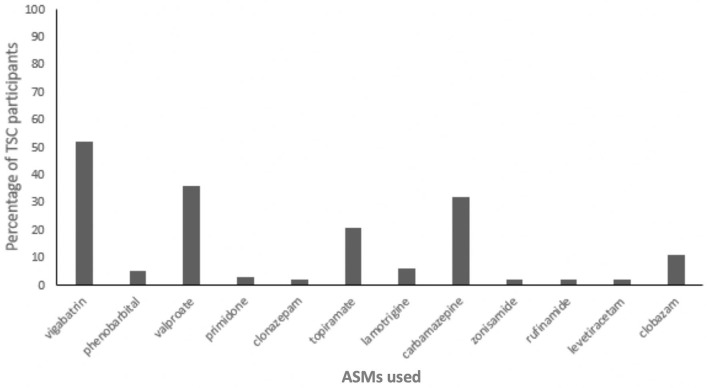
Proportion of Tuberous Sclerosis Complex (TSC) participants using antiseizure medications (ASMs) (*n* = 66) (38, 41).

Data relating to ASM use was not commonly provided in the studies included in this review. Where reported, the mean number of ASMs per patient with TSC ranged from 1.46 to 3.95 (12, 13, 18, 38). Combination treatment with two ASMs or more was common and, where reported, the number of TSC patients using polytherapy ranged from 52 to 100% (20, 21, 25, 29, 38). Common ASMs included valproic acid, carbamazepine, topiramate, lamotrigine, and vigabatrin (25, 38, 41). Only two of the reviewed studies reported individual use of ASMs among TSC patients, and these data are summarized in Figure 2.

Early treatment with ASMs may be of importance, since better long-term epileptic encephalopathy outcomes were reported in those treated early in a randomized trial of early vs. later treatment with vigabatrin (41). In general, studies should distinguish between early and later treatment of epilepsy in TSC, considering that later treatment of seizures in TSC is often disappointing and research reports that the development of ID is predicted by the number of ASMs used (potentially related to delay in effective treatment) to treat epilepsy in children with TSC (38).

The studies in our review with data on individuals with uncontrolled epilepsy reported these to represent 14–100% of patients with a history of epilepsy, with the majority of studies reporting >40% of the epilepsy population still having seizures (Table 2). Although these data suggest a greater proportion of TSC patients with difficult-to-treat epilepsy than is typical of a general population, the bias in study participation remains a caveat to such speculation.

Three studies evaluated the effects of an mTOR kinase inhibitor, everolimus, which can be used to reduce tumor size (44, 45, 47, 48). The first, a three-armed randomized trial in Japan (*n* = 29), reported adjunctive everolimus treatment to significantly reduce seizure frequency in TSC patients with refractory epilepsy, with a trend for improvements in ASD symptoms (45). A similar finding was reported in a small case study evaluating everolimus for refractory epilepsy in six TSC patients with refractory epilepsy (44). This second study also reported improvement in ASD symptoms, such as social contact, language, and repetitive behavior (44). However, the third study—a recent randomized controlled trial conducted in the Netherlands including 32 children with TSC—found no benefit of everolimus on cognitive or neuropsychological functioning, or autism traits, in comparison with placebo (47). In this third study, age at enrollment was high—the median age was 11.5 years for patients on placebo and 12.2 years for patients on everolimus—therefore, firm conclusions cannot be drawn (47). However, early treatment with everolimus might be required for improvement in features such as social contact, language and repetitive behavior; there is a need for formal studies to determine whether this is the case.

## Discussion

Based on the 36 articles included in this review, our findings were consistent with previous reports of high rates of epilepsy in patients with TSC (5). Interestingly, epilepsy was reported in 83.6% of patients with TSC in an international TuberOus SClerosis registry to increase disease Awareness (TOSCA); however, data on the prevalence of ASD in this population were not reported (56). The prevalence of autism in patients with TSC in the subjects included in this review is high, but is consistent with previous estimates of syndromic ASD in TSC (14, 15, 57).

The risk of autism is increased by early onset seizures (18, 28, 31, 35) and by DD and ID (28), which in turn have been associated with early onset epilepsy and infantile spasms (28, 31, 32, 35, 38). Existence of phenotypic variability should be acknowledged: TSC is also associated with high-functioning autism, normal intelligence, hypercalculia, and drug-resistant epilepsy with an EEG pattern characterized by hypsarrhythmia and electrical status epilepticus during sleep (58).

The relationship between TSC, epilepsy, and ASD is highly complex. A poor prognosis of epilepsy outcomes is largely reported to be exacerbated by ASD (18, 21, 31). An additive neuroanatomical impact of TSC, epilepsy, and autism has been proposed that is predominantly evident in white-matter pathways (36, 37), supporting the association between autism, epilepsy, and DD/ID in patients with TSC.

Evidence suggests both epilepsy and autism are linked with mutations on the *TSC1* and *TSC2* genes. Mutations in the *TSC2* gene are more prevalent in association with epilepsy and autism (18, 22, 29, 38, 40, 50). Early genotyping may, therefore, help identify TSC patients at increased risk of poorer long-term outcomes.

In terms of autism, neuroimaging studies report that tuber features, such as larger size or increased number of cyst-like tubers, are associated with increased risk (22, 26). It is also demonstrated that diffusion imaging abnormalities correlate with reduced myelination in TSC patients (59) and the effect of mTOR overactivation on white matter might be modified by pharmacological inhibition (60). Moreover, TSC patients with autism have been documented to have a reduction of fractional anisotropy in different white-matter regions, and this happens over the first 2 years of life (61). Since size, type, and location of tubers influence the longer-term risk of autism and epilepsy in TSC, early characterization of such features could assist in determining the focus of early intervention.

Cells in the central nervous system express TSC1 and TSC2 proteins throughout childhood and into adulthood. These proteins help regulate myelination, axon guidance, and dendritic arborization, promoting normal synaptic formation and function (37). Dysregulation of the neurotransmission of GABA, resulting from genetic mutations of TSC, has previously been argued to underlie development of epilepsy and autism in this population (5). Limited evidence suggests that treatment with everolimus, particularly if commenced early, may improve epilepsy outcomes and reduce the risk of autism in TSC patients (44, 45). Data coming from the EXIST-3 trial confirm that adjunctive everolimus might reduce seizure frequency in pediatric patients with treatment-refractory seizures associated with tuberous sclerosis complex also in patients younger than 6 years (62). However, these findings were based on few trials and contradictory evidence also exists (47). Additional research into alternative treatment strategies and an increased focus on the longer-term outcomes would help elucidate whether size, type, and location of tubers influence the longer-term risk of autism and epilepsy in TSC.

**Figure 3:**
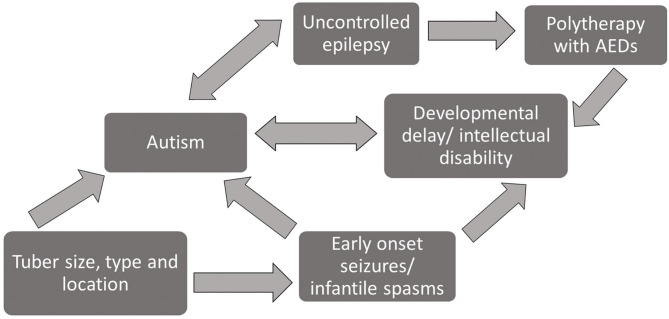
Diagram of interrelationship between phenotypic profiles and prognostic risk factors among patients with TSC.

Early treatment with ASMs to control epilepsy is reported to improve longer-term epilepsy outcomes (41), and controlled epilepsy is associated with reduced symptoms of autism (44, 45). ID and DD are in turn associated with increased presence of autism (31, 34), so the number and choice of ASMs in infants with TSC needs to be managed with care. Figure 3 is a diagrammatic overview of the complex relationship between the phenotypic features of TSC and polytherapy treatment with ASMs based on the evidence reviewed here.

## Limitations

Although we identified 36 articles reporting autism and epilepsy in TSC, only approximately half of these articles indicated which patients were experiencing either of these comorbid conditions. Very few of the included studies summarized the potential prognostic features of patients with all three conditions (TSC, epilepsy, and autism). This review has therefore identified a need for future studies to focus on common associative factors.

A second limitation was that, because the studies were conducted in different settings across different countries, practices were not standardized with respect to identification of TSC, epilepsy, and—above all—autism. Different diagnostic criteria were used to identify patients with TSC according to the clinical practice of the country or region. Likewise, the tools used to define the presence of autism varied considerably. In some cases, the diagnosis of autism was not confirmed, but relied on reports from parents and caregivers. In populations that only focused on very young infants, in whom a clinical diagnosis of autism was not possible, the conclusions regarding risk of autism were based on autistic features, which do not necessarily indicate a later clinical outcome.

The methodological approaches of the included articles also varied widely and ranged from small clinical series to large retrospective studies, each with differing strengths and limitations. One of the challenges of establishing a representative sample of individuals with TSC is the rarity of the disease. The TSC populations within the included articles ranged from infants to adults, sometimes within the same study. Consequently, the core features of TSC and age of onset of the conditions may not have been reliable.

Lastly, the quality of the available data does not allow a meaningful review to be performed.

## Conclusions

Early onset epilepsy, frequently represented by epileptic encephalopathy, can be considered one of the risk factors for ID in TSC patients. However, the role of genetic variations should be highlighted as the major player in determining both epilepsy and intellectual disability due to mTOR overactivation (63).

In terms of further defining the prognostic features of epilepsy and autism within TSC, large prospective studies, such as TACERN or those conducted by the EPISTOP group (34, 51, 64), are helping to identify early biomarkers for treatment.

The prevalence of autism and epilepsy in TSC is much higher than that in the general population, both alone and as comorbid features. We summarized the phenotypic, genetic, and neurological risk factors for the association of autism and epilepsy in TSC patients from available data, but the inherent limitations of the source studies should be noted.

The relationship between these three conditions is complex. Early identification of the risk factors, together with early use of m-TOR inhibitors might be a priority to optimize favorable outcomes in this vulnerable population.

## Author Contributions

NS conceptualized and designed the study, drafted the initial manuscript, supervised data collection, and reviewed and revised the manuscript. NP, MT, and LD designed the data collection instruments, collected data, carried out the initial analyses, and reviewed and revised the manuscript. AF and RM collected data, carried out the initial analyses, and reviewed and revised the manuscript. FV and PC conceptualized and designed the study and critically reviewed the manuscript for important intellectual content. All authors contributed to the article and approved the submitted version.

## Conflict of Interest

NS has received support from Livanova and Biomarin, and has served as a paid consultant for Livanova. PC has served as a paid consultant for Novartis. FV has served as paid consultant for Zogenix, Eisai, and GW Pharma. MT has served as paid consultant for Biomarin. The remaining authors declare that the research was conducted in the absence of any commercial or financial relationships that could be construed as a potential conflict of interest.

## Abbreviations

ADC: apparent diffusion coefficient
ASM: antiseizure medication
ASD: Autism Spectrum Disorder
EEG: electroencephalogram
DD: developmental delay
FA: fractional anisotropy
GABA: gamma-amino-butyric acid
ID: intellectual disability
MRI: magnetic resonance imaging
PDD: pervasive developmental disorder
QUIPS: Quality in Prognosis Strategy
TSC: Tuberous Sclerosis Complex.
